# Optimal Standardized Ileal Digestible Total Sulfur Amino Acids to Lysine REQUIREMENTS Are Increased in Nursery Pigs Raised under Antibiotic-Free Feeding Regime

**DOI:** 10.3390/ani11113143

**Published:** 2021-11-03

**Authors:** Ping Ren, Ferdinando Almeida, Uislei Orlando, Marcio Gonçalves, Deana Hancock, Mercedes Vazquez-Añón

**Affiliations:** 1 Novus International, Inc., St. Charles, MO 63304, USA; Ferdinando.Almeida@ourofino.com (F.A.); Deana.Hancock@novusint.com (D.H.); Mercedes.Vazquez@novusint.com (M.V.-A.); 2 Genus PIC North America, Hendersonville, TN 37075, USA; Uislei.Orlando@genusplc.com (U.O.); Marcio@swineit.com (M.G.)

**Keywords:** antibiotics-free, 2-hydroxy-4-(methylthio)butanoic acid, lysine, growth performance, pigs, total sulfur amino acid

## Abstract

**Simple Summary:**

Total sulfur amino acids play a critical role in numerous biological functions, including antioxidative status and immunity, as well as protein synthesis. Weaning pigs commonly face multiple stressors which can impair their gut integrity and growth performance. Antibiotic removal from the diets in weaning pigs can stimulate immune response and divert nutrients from growth to optimize immune function. The objective of the current two studies was to determine the optimal ratio of the standardized ileal digestible (SID) total sulfur amino acid to lysine (TSAA:Lys) in nursery pigs under an antibiotics-free feeding regime. The results demonstrated that the optimal SID TSAA:Lys for nursery pigs raised without antibiotics during the first 21 d post-weaning was 62 to 72% in terms of growth performance, whereas the optimal SID TSAA:Lys was approximately 58% in terms of growth performance in the late nursery phase.

**Abstract:**

This study aimed to investigate the effect of increasing the standardized ileal digestible (SID) total sulfur amino acid to lysine (TSAA:Lys) on the growth performance of nursery pigs raised with or without antibiotics (AGP) and to determine the optimal SID TSAA:Lys in nursery pigs raised without AGP. In Exp. 1, 924 nursery pigs (7.9 ± 1.3 kg), blocked by initial BW and sex, were randomly allotted to one of six treatments, with seven pens per treatment and twenty-two pigs per pen. The treatments were arranged in a 2 × 3 factorial design, with two AGP levels (0 or 50 mg/kg Carbodox) and three levels of SID TSAA:Lys (51.0, 58.5 or 66.0%). In Exp. 2, 990 weaned piglets (5.1 ± 0.9 kg), blocked by initial BW and sex, were randomly allotted to one of five dietary treatments (SID TSAA:Lys at 51, 58, 65, 72 or 79%) in the absence of AGP, with nine pens per treatment and twenty-two pigs per pen. Competing heteroskedastic models including broken-line linear (BLL), broken-line quadratic (BLQ), and quadratic polynomial (QP) were fitted for the growth performance data to estimate the optimal TSAA:Lys. In Exp. 1, AGP supplementation increased (*p* < 0.05) ADG and ADFI during the 21 d period. Increasing SID TSAA:Lys in the diets with AGP did not affect growth performance; however, increasing SID TSAA:Lys in the diets without AGP resulted in a linear increase (*p* < 0.05) in ADG and G:F. In Exp. 2, the best-fitting models for ADG and G:F from d 0 to 21 post-weaning were BLL, which yielded the optimal SID TSAA:Lys of 62% and 72%, respectively. The best-fitting models for ADG and G:F from d 21 to 42 post-weaning were BLL, which yielded the optimal SID TSAA:Lys of 59% and 58%, respectively. In conclusion, SID TSAA to Lys requirements under an antibiotic-free feeding regime during the first 21 d post-weaning were 62% and 72% in terms of ADG and G:F, respectively, whereas an SID TSAA:Lys of approximately 58% was required to maximize ADG and G:F for the late nursery phase.

## 1. Introduction

Total sulfur amino acids (TSAA), composed of methionine (Met) and cysteine (Cys), are considered as the second or third most limiting amino acid in the diets of nursery pigs [1]. Methionine can be converted to Cys via cystathionine through the transmethylation and trans-sulfuration pathways [2]. Moreover, the function of protein synthesis, Met, has numerous biological functions, including being a precursor for methyl donors, glutathione and taurine [3]; an antioxidative effect [4], and an adaptive immunity enhancement [5,6]. These functional roles of Met are critical for the growth development and health status of pigs [7].

Antibiotic growth promoters (AGP) have been used in monogastric diets for many decades to control disease and improve growth performance. With the removal of AGP in the diets, the lack of enteric pathogen control stimulates an immune response and therefore diverts nutrients away from aiding growth to meet the immunity function [8]. It was demonstrated that the tryptophan (Trp) or threonine (Thr) requirements of the pigs were higher when pigs that were fed diets without antibiotics compared with diets with antibiotics [9,10]. Additionally, the TSAA requirement was also increased in nursery pigs challenged with a pathogenic strain of E. coli [11]. Traditionally, the TSAA requirements for nursery pigs were determined when the antibiotics were used in the diets or water. With the ban of AGP in their diets, there is a need to establish the TSAA requirement for nursery pigs fed diets without AGP. To our best knowledge, no studies have reported the TSAA requirement for nursery pigs under an antibiotics-free regime. It was hypothesized that the pigs fed diets without AGP would have an increased immune stimulation, and thus a higher TSAA requirement. Two studies were conducted to investigate the effect of increasing the standardized ileal digestible (SID) total sulfur amino acid to lysine ratio (TSAA:Lys) on the growth performance of nursery pigs fed diets with or without AGP, and to determine the optimal SID TSAA:Lys in nursery pigs fed diets without AGP.

## 2. Materials and Methods

### 2.1. Experiment 1

The experiment was conducted at a commercial nursery research facility in central Missouri, USA. A total of 924 PIC 337 × 1050 nursery pigs at d 35 of age (BW = 7.9 ± 1.3 kg; PIC, Hendersonville, TN, USA) were used in this study. Pigs were weaned at d 21 of age (BW = 5.7 ± 1.1 kg) in a commercial sow farm and then moved to the commercial nursery research facility. Two common mash diets were fed to all pigs from d 0 to 7 and from d 7 to 14 (Table 1) post-weaning, respectively. On 14 d post-weaning, pigs were weighed and randomly allotted to 1 of 6 treatments according to a randomized complete block design, blocking by initial BW and balanced by sex in each pen, with 7 pens per treatment and 22 pigs per pen. Pigs were fed experimental diets from 14 to 35 d post-weaning. Two basal diets were formulated with or without the addition of 1.0% Mecadox 2.5 (50 mg/kg Carbodox; Phibro Animal Health Corp., Ridgefield Park, NJ). The basal diets were corn–soybean, meal-based diets formulated to meet or exceed NRC [12] recommendations for 11–25 kg pigs, except for SID Lys and SID TSAA. In the basal diets, the SID Lys was set for 1.10%, which were marginally deficient based on the estimated requirement for pigs under the proposed conditions (i.e., genotype and environment) for this study, whereas the SID TSAA was set at 0.55% (50% of SID Lys). Four additional diets were prepared by supplementing the basal diets with 2 levels of dry calcium salt of D, L-2-hydroxy-4-(methylthio)butanoic acid (HMTBa-Ca, 84% methionine activity, MHA, Novus International, Inc., St. Charles, MO) to produce diets with 0.64% and 0.73% SID TSAA (58.5 and 66% of SID TSAA:Lys, respectively; Table 1). The experimental diets were in mash form.

### 2.2. Experiment 2

The experiment was conducted at a commercial nursery research facility in central Missouri, USA. A total of 990 PIC 337 × 1050 weaned piglets (BW = 5.1 ± 0.9 kg; PIC, Hendersonville, TN, USA) were used in this study. A three-phase nursery feeding program (d 0–7, 7–21, and 21–42 post-weaning) was implemented in this study. No antibiotics were used in the diets and water. Pigs received their experimental diets in all three phases. Phase 1 and 2 diets were pelleted, whereas phase 3 diets were in mash form.

At the initiation of this study, piglets were weighed individually and allotted to 1 of 5 dietary treatments according to a randomized complete block design, blocked by initial BW and balanced by sex in each pen, with 9 pens per treatment and 22 pigs per pen. Five diets for each phase were formulated to meet the nutrient requirement of nursery pigs by PIC recommendation [13], except that SID Lys concentrations in each phase were reduced by 10% from the requirement to create Lys marginal deficiency, whereas SID TSAA to Lys ratios were set at 51%, 58%, 65%, 72%, and 79% by addition of HMTBa-Ca for 5 respective diets (Table 2).

Pen weights were measured at the initiation (d 0), and at the end of each phase (d 7, 21 and 42). Feed addition to each feeder was recorded each time when the feed was added to the feeder. At the end of each phase, remaining feed in the feeder was weighed. The ADG, ADFI, and G:F were calculated for each phase and the entire period.

### 2.3. Statistical Analysis

SAS^®^ 9.4 (SAS Inst. Inc., Gary, NC, USA) was used for all data analyses. Pen served as the experimental unit. The LSMEANS statement was used to calculate the least square means. Tukey–Kramer adjustment was used for multiple comparisons of the least square means. Pooled SEM was calculated for each measurement. A probability of *p* ≤ 0.05 was considered as significant and 0.05 < *p* ≤ 0.10 was declared as a trend.

In Experiment 1, a GLIMMIX procedure was used to analyze data. Data were analyzed by two-way ANOVA. Antibiotics, SID TSAA:Lys, and their interactions were considered as the fixed effect, whereas block was the random effect. Polynomial contrasts were conducted to test linear, quadratic, and cubic effect of increasing SID TSAA to Lys ratios on growth performance.

In Experiment 2, GLIMMIX procedure was used to analyze data. Diet was considered as the fixed effect, whereas block was the random effect. Polynomial contrasts were conducted to test linear, quadratic and cubic effect of increasing SID TSAA to Lys ratios on growth performance. A broken-line linear ascending model (BLL), a broken-line quadratic ascending model (BLQ), and a quadratic polynomial model (QP) were fitted to the data for growth performance data to estimate the optimal TSAA to Lys ratios [14]. Correlated data structure and heterogenous error variance were accounted for in the three models. The best model was selected based on the maximum likelihood-based Bayesian information criterion (BIC), with BIC values greater than 2 considered a significant improvement in fit [15]. For the models with similar BIC values, the model with the narrowest 95% confidence interval (CI) was considered to be the best model.

## 3. Results

### 3.1. Experiment 1

**Figure 1:**
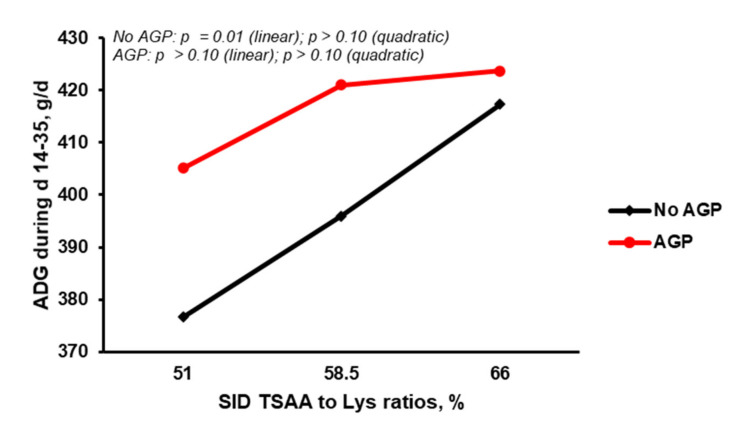
Effect of antibiotic growth promoter (AGP) and standardized ileal digestible (SID) total sulfur amino acids to lysine ratio (TSAA:Lys) on average daily gain of nursery pigs from d 14 to 35 (orthogonal contrast analysis).

**Figure 2:**
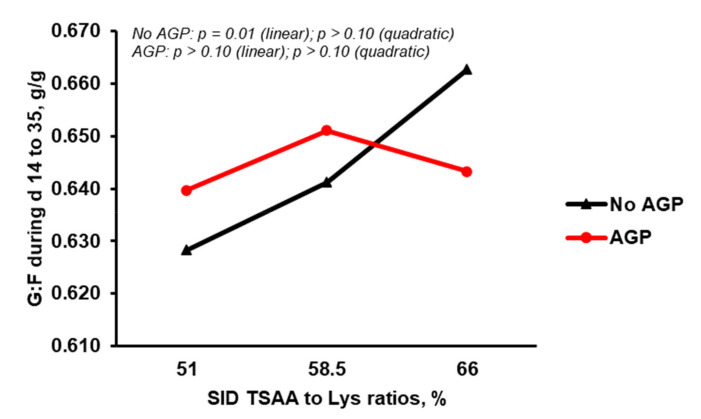
Effect of antibiotic growth promoter (AGP) and standardized ileal digestible (SID) total sulfur amino acids to lysine ratio (TSAA:Lys) on gain to feed ratio of nursery pigs from d 14 to 35 (orthogonal contrast analysis).

The pigs fed diets supplemented with AGP had a higher (*p* < 0.05) final BW, ADG, and ADFI compared with those fed diets without AGP (Table 3). Under the condition of no AGP in the diets, increasing the SID TSAA:Lys from 51 to 66% resulted in a linear (*p* = 0.01) increase in ADG (Figure 1) and G:F (Figure 2). However, increasing SID TSAA:Lys from 51 to 66% did not affect (*p* > 0.10) ADG (Figure 1) and G:F (Figure 2) in the pigs fed diets containing AGP.

### 3.2. Experiment 2

#### 3.2.1. SID TSAA to Lys Ratios on Growth Performance of Nursery Pigs Raised without Antibiotics

Increasing the SID TSAA:Lys tended to increase BW on d 21 (quadratic; *p* < 0.10), BW on d 42 (linear; *p* < 0.10; Table 4), ADG from d 0 to 21 (quadratic; *p* < 0.10), ADFI from d 7 to 21 (quadratic; *p* < 0.10), and ADFI from d 0 to 42 (quadratic; *p* < 0.10). Similarly, ADG from d 7 to 21 and ADG from d 0 to 42 were increased in quadratic (*p* < 0.05) and linear (*p* < 0.05) manners with increase in SID TSAA:Lys, respectively.

#### 3.2.2. Estimation of Optimal Ratio of SID TSAA to Lys in Nursery Pigs Raised without Antibiotics

Three different dose–response models were fitted to estimate the optimal ratio of SID TSAA to Lys in nursery pigs under ABF. The average daily gains and G:F from d 0 to 21 and d 21 to 42 were used as the response criteria for model estimation.

**Figure 3:**
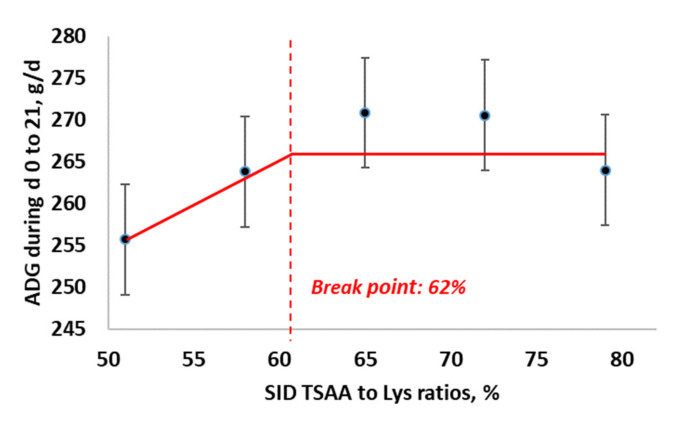
Broken-line linear ascending model for estimation of optimal SID TSAA to Lys ratio in nursery pigs under ABF for average daily gain from d 0 to 21.

For the average daily gain from d 0 to 21, the BLL model showed the best fit (BIC = 406.9), compared with the QP (BIC = 444.28) and BLQ (BIC = 409.1) models. The maximum ADG from d 0 to 21 was obtained as 62% SID TSAA to Lys (95% CI: [42, 82%]). The estimated regression equations for the best fitting BLL model (Figure 3) were:

$$\begin{array}{c} \mathrm{ADG}\;=268.47-115.79\times \left(0.62-\mathrm{TSAA}\;\mathrm{to}\;\mathrm{Lys}\right),\;\mathrm{if}\;\mathrm{SID}\;\mathrm{TSAA}\;\mathrm{to}\;\mathrm{Lys}\;<0.62\\ \mathrm{ADG}=268.47,\;\mathrm{if}\;\mathrm{SID}\;\mathrm{TSAA}\;\mathrm{to}\;\mathrm{Lys}\;\geq \;0.62 \end{array}$$

**Figure 4:**
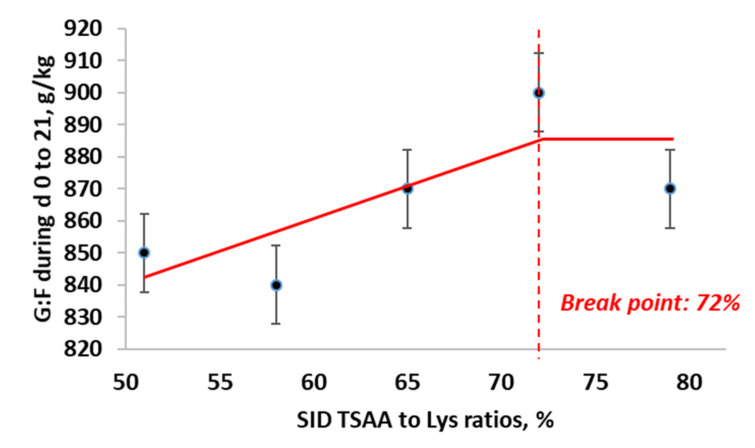
Broken-line linear ascending model for estimation of optimal SID TSAA to Lys ratio in nursery pigs under ABF for gain to feed from d 0 to 21.

For the gain to feed ratio from d 0 to 21 (g/kg), BLL, BLQ, and QP models all showed similar fits (BIC = 477.4, 477.2 and 477.9, respectively). However, BLL model has a narrower 95% confidence interval compared with the other two models, therefore BLL model was selected as the best model. The maximum gain to feed ratio from d 0 to 21 was obtained at 72% SID TSAA to Lys (95% CI: [71.8, 72.2%]). The estimated regression equations for the best fitting BLL model (Figure 4) were:

$$\begin{array}{c} G:F\;=883.97-206.57\times \left(0.72-\mathrm{TSAA}\;\mathrm{to}\;\mathrm{Lys}\right),\;\mathrm{if}\;\mathrm{SID}\;\mathrm{TSAA}\;\mathrm{to}\;\mathrm{Lys}\;<0.72\\ G:F=883.97,\;\mathrm{if}\;\mathrm{SID}\;\mathrm{TSAA}\;\mathrm{to}\;\mathrm{Lys}\;\geq \;0.72 \end{array}$$

**Figure 5:**
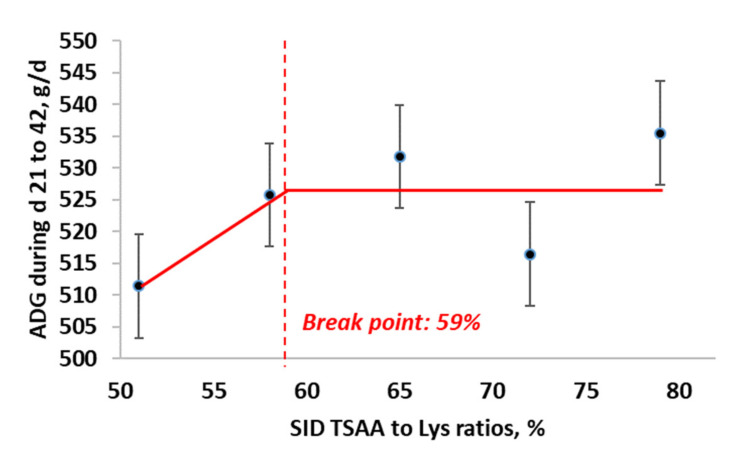
Broken-line linear ascending model for estimation of optimal SID TSAA to Lys ratio in nursery pigs under ABF for average daily gain from d 21 to 42.

For average daily gain from d 21 to 42, BLL model showed the best fit (BIC = 428.6), compared with QP (BIC = 429.5) and BLQ (BIC = 430.8) models. The maximum ADG from d 21 to 42 was obtained at 59% SID TSAA to Lys (95% CI: [47, 70%]). The estimated regression equations for the best fitting BLL model (Figure 5) were:

$$\begin{array}{c} \mathrm{ADG}\;=527.13-205.05\times \left(0.59-\mathrm{TSAA}\;\mathrm{to}\;\mathrm{Lys}\right),\;\mathrm{if}\;\mathrm{SID}\;\mathrm{TSAA}\;\mathrm{to}\;\mathrm{Lys}\;<0.59\\ \mathrm{ADG}=527.13,\;\mathrm{if}\;\mathrm{SID}\;\mathrm{TSAA}\;\mathrm{to}\;\mathrm{Lys}\;\geq \;0.59 \end{array}$$

**Figure 6:**
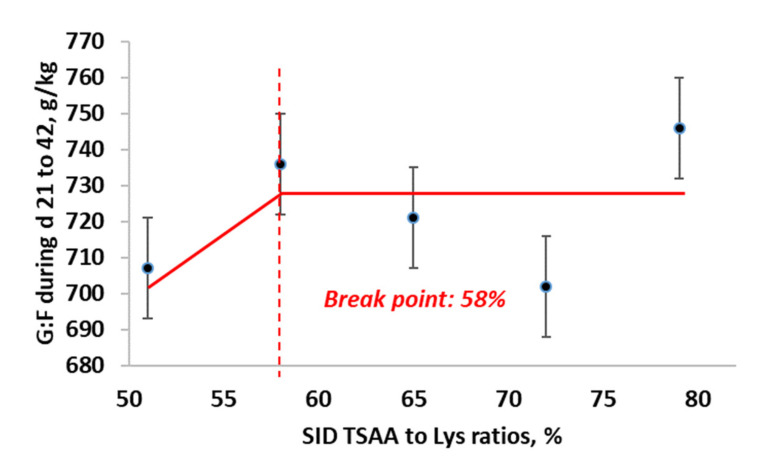
Broken-line linear ascending model for estimation of optimal SID TSAA to Lys ratio in nursery pigs under ABF for gain to feed from d 21 to 42.

For the gain to feed ratio from d 21 to 42 (g/kg), the BLL model showed the best fit (BIC = 472.2), compared with the QP (BIC = 473.03) and BLQ (BIC = 474.4) models. The maximum gain to feed ratio from d 21 to 42 was obtained at 58% SID TSAA to Lys (95% CI: [57.8, 58.2%]). The estimated regression equations for the best fitting BLL model (Figure 6) were:

$$\begin{array}{c} G:F\;=724.17-275.55\times \left(0.58-\mathrm{TSAA}\;\mathrm{to}\;\mathrm{Lys}\right),\;\mathrm{if}\;\mathrm{SID}\;\mathrm{TSAA}\;\mathrm{to}\;\mathrm{Lys}\;<0.58\\ G:F=724.17,\;\mathrm{if}\;\mathrm{SID}\;\mathrm{TSAA}\;\mathrm{to}\;\mathrm{Lys}\;\geq \;0.58 \end{array}$$

## 4. Discussion

### 4.1. Antibiotics Growth Promoter on Growth Performance and Amino Acid Requirement

Antibiotic growth promoters have been used in monogastric diets for decades to control disease and improve growth performance. Cromwell [16] demonstrated that AGP improved the growth rate and feed efficiency by 16.4 and 6.9% in nursery pigs by conducting a meta-analysis based on 1000 pig growth studies, respectively. Similarly, in a meta-analysis based on 183 broiler studies, Cardinal et al. [17] reported that AGP improved weight gain and feed efficiency from d 1 to 42 by 3.9% and 3.5%, respectively. The weight gain response by AGP in our first study was consistent with previous findings, even though the feed efficiency benefit of AGP was not observed. The exact modes of action regarding how AGP can improve growth performance are not well understood. It was demonstrated that AGP supplementation in the diets could reduce total number of gut bacteria and the number of bacteria species [18], reduce the thickness of the gut wall and villus lamina propria [19], increase amino acid absorption by enhancing jejunum and ileum amino acid transporters [20,21], reduce inflammation, and change whole-body protein metabolism [22]; however, it may not affect the intestinal permeability and fiber fermentation [22,23].

The phenomenon that increasing SID TSAA:Lys from 51 to 66% in the presence of AGP did not impact growth performance in the current study for pigs from 8 to 16 kg may be due to the fact that SID TSAA:Lys at 51% might already meet the requirement with the presence of AGP. However, with the removal of AGP from the diets, the lack of enteric pathogen control could stimulate immune system activation, and thus more nutrients could be diverted from growth to support immune function [8]. As a result, the optimal SID TSAA:Lys was increased when AGP was not present in the diets. Similarly, the Trp or Thr requirements of the pigs were higher when pigs were fed diets without AGP compared with diets containing antibiotics [9,10]. It was also shown that SID TSAA:Lys increased in nursery pigs challenged with a pathogenic strain of *E. coli* [11].

### 4.2. Function of Methionine and Increased Methionine Requirement without AGP

Increasing evidence suggests that the sulfur amino acids, Met and Cys, are essential in numerous biological functions and diseases, as well as their role in protein synthesis. For instance, Met plays an important role in cellular antioxidant functions. Methionine can be converted to Cys via cystathionine through the transmethylation and trans-sulfuration pathways [2]. Cysteine plays an important role in cellular redox function and managing oxidative stress in the intestine [24,25]. Cysteine is a constituent amino acid of the tripeptide glutathione (γ-Glu-Cys-Gly), the major cellular antioxidant in mammals, and also serves as a precursor for the synthesis of taurine, sulfate, and coenzyme A [2]. Glutathione has a critical role in cellular detoxification against oxidative and chemical injury [26]. Additionally, Met can function as an antioxidant agent [27]. Numerous reactive oxygen species react readily with methionine residues in proteins to form methionine sulfoxide, which can be catalyzed by methionine sulfoxide reductase back into methionine, therefore scavenging the reactive oxygen species [4,27]. Maintaining a normal redox status is particularly important for intestinal epithelial cells, which are constantly exposed to high levels of oxidative stress due to high rates of oxidative metabolism [28]. Studies in intestinal epithelial cells indicate that the increased oxidative stress and redox imbalance can suppress cell proliferation and induce apoptosis [29,30,31].

Studies demonstrated that about 20 to 30% of dietary Met intake was metabolized in the gastrointestinal tract of infant pigs, and that much of the Met used by the gut was metabolized via the transmethylation and trans-sulfuration pathways [32,33]. As a result, Met requirement was about 30% higher in the enterally fed than in the parenterally fed piglets, reinforcing the important first-pass metabolic demand of the gut [33]. Therefore, an adequate Met intake is required to maximize the growth performance of pigs. Bauchart-Thevret et al. [7] reported that sulfur amino acid deficiency upregulated intestinal methionine cycle activity and suppressed epithelial growth in neonatal piglets. It was also demonstrated that nursery pigs fed diets without antibiotics had an increased oxidative stress compared with those fed diets with antibiotics [34]. It was probable that the piglets fed diets containing SID TSAA:Lys between 51.0 to 58.5% without AGP in our first study were deficient in Met; therefore, a higher level of SID TSAA:Lys was required to optimize the growth performance in nursery pigs fed diets without AGP, which was confirmed in the second study.

### 4.3. Optimal SID TSAA:Lys in Nursery Pigs without AGP

Methionine is the second or third limiting amino acid in corn–soybean, meal-based diets for pigs [1]. The supplemental types of Met used in animal diets are DL-Met, L-Met and HMTBa [1,35]. Numerous studies showed that HMTBa had a similar or better absorption rate compared with DL-Met and L-Met [36,37], which was mainly driven by the passive diffusion pathway for HMTBa versus the energy-dependent pathways for DL-Met and L-Met. Previous studies also demonstrated that HMTBa had similar bioefficiency compared with DL-Met and L-Met in pigs [1,38], despite some inconsistent findings from other studies [39,40]. Therefore, the optimal levels of SID TSAA:Lys in pigs fed diets containing one of these three Met sources may be similar.

It is reported that weaned pigs exhibited an increased intestinal permeability that was most pronounced at 24 h post-weaning and then gradually declined over the first 2 weeks post-weaning, compared with age-matched and non-weaned littermates [41]. At the same time, weaning in piglets was associated with an upregulation of inflammatory cytokines in the intestine [42]. It was also shown that sulfur amino acid played an important role in the innate immune system and stimulating humoral and cell-mediated immune responses [43,44]. To the best of our knowledge, this was the first study to report the increased SID TSAA:Lys requirements for nursery pigs fed diets without AGP. The optimal SID TSAA:Lys was 62% and 72% for ADG and G:F during the first 21 d post-weaning, respectively, which may be explained by the fact that the weaning stress and the associated immune system stimulation would increase sulfur amino acid requirements to manage oxidative stress and inflammation. Similarly, Capozzalo et al. [11] demonstrated that ADG and G:F were optimized at 71% and 68% in the weaning pigs challenged with enterotoxigenic *E. coli*, respectively. It was also shown that 65.9% and 63.4% SID TSAA:Lys were required to optimize the jejunum villus height and plasma urea nitrogen in weaning pigs under unclean sanitary conditions [45]. In the late nursery period (from d 21 to 42), due to the improved intestinal integrity and immune systems, SID TSAA:Lys of approximately 58% was needed to optimize growth performance.

## 5. Conclusions

Nursery pigs fed diets containing no AGP had a greater requirement of SID TSAA:Lys than those fed diets containing AGP. The optimal SID TSAA:Lys for ADG and G:F in nursery pigs during the first 21 d post-weaning were 62% and 72%, respectively, indicating that SID TSAA:Lys under an antibiotic-free regime in the early nursery period was 13 to 31% greater than the NRC (2012) recommendation. However, an SID TSAA:Lys of approximately 58% was required to maximize ADG and G:F for the late nursery phase, indicating that SID TSAA:Lys under an antibiotic-free regime in the late nursery period was 5% higher than the NRC (2012) recommendation.

## Figures and Tables

**Table 1:** Ingredient and nutrient composition of common diet in phases 1 and 2, and basal diets for phase 3 with 51% standardized ileal digestible total sulfur amino acid to lysine ratio (Experiment 1; as-fed basis).

	P1	P2	P3	
**Ingredients**			**no AGP-51% ^1^**	**AGP-51% ^1^**
Corn, yellow dent	38.71	56.70	64.48	62.77
Soybean meal, 47.5% CP	15.00	25.00	31.00	31.00
Whey, dried	30.00	8.20	0.00	0.00
Plasma spray-dried	7.00	0.00	0.00	0.00
Fish meal, menhaden	5.15	7.00	0.00	0.00
L-lysine HCl	0.19	0.26	0.21	0.21
L-threonine	0.04	0.08	0.11	0.12
MHA ^2^	0.19	0.16	0.00	0.00
L-tryptophan	0.02	0.04	0.00	0.03
Choice white grease	1.83	0.32	1.00	1.70
Monocalcium phosphate 21%	0.17	0.25	0.98	0.97
Limestone	0.55	0.72	1.11	1.10
Salt	0.25	0.49	0.61	0.60
Mecadox 2.5 (0.55%)	0.00	0.00	0.00	1.00
Vitamin and mineral premix ^3^	0.50	0.50	0.50	0.50
Zinc oxide, 72% Zn	0.40	0.28	0.00	0.00
Total	100.00	100.00	100.00	100.00
Nutrient composition				
ME ^4^, kcal/kg	3395	3307	3402	3402
CP ^4^, %	23.90	21.80	20.30	20.30
SID ^5^, %				
Lys	1.47	1.31	1.10	1.10
Thr	0.88	0.79	0.74	0.74
Met	0.47	0.48	0.28	0.28
Met+Cys	0.85	0.76	0.56	0.56
Trp	0.29	0.26	0.22	0.22
Ca, %	0.85	0.85	0.75	0.75
STTD ^6^ P, %	0.53	0.40	0.33	0.33

^1^ Fifty-one percent represented standardized ileal digestible (SID) total sulfur amino acid to lysine ratio (TSAA:Lys). The experimental diets with 58.5% and 66% SID TSAA:Lys were made by adding 0.10% and 0.20% MHA in the basal diets, respectively. ^2^ MHA is dry calcium salt of D, L-2-hydroxy-4-(methylthio) butanoic acid (84% Met activity, MHA, Novus International, Inc., St. Charles, MO, USA). ^3^ Vitamin and mineral premix supplied the following nutrients per kilogram of diets: vitamin A, 11,000 IU; vitamin D, 1760 IU; vitamin E, 83.6 IU; vitamin K, 5.5 mg; thiamine, 3.52 mg; riboflavin, 13.2 mg; niacin, 70.4 mg; pantothenic acid, 39.6 mg; pyridoxine, 7.04 mg; folic acid, 1045 µg; biotin, 275 µg; vitamin B12, 55 µg; Zn, 120 mg as zinc sulphate; Cu, 12 mg as copper; Mn, 30 mg as manganese sulphate; Fe, 80 mg as ferrous sulphate; I, 0.4 mg as ethylenediamine dihydriodide; and Se, 0.3 mg as sodium selenite. ^4^ ME and CP represented metabolizable energy and crude protein, respectively. ^5^ SID = standardized ileal digestible. ^6^ STTD = standardized total tract digestible.

**Table 2:** Ingredient and nutrient composition of phase 1 (d 0–7), phase 2 (d 7–21), and phase 3 (d 21–42) basal diets with 51% standardized ileal digestible total sulfur amino acid to lysine ratio (Experiment 2; as-fed basis).

Ingredients	P1-51% ^1^	P2-51% ^2^	P3-51% ^3^
Corn, yellow dent	20.74	36.39	63.15
Oat groats	20.00	15.00	0.00
Soybean meal, 47.5% CP	15.00	20.00	31.00
HP300 ^4^	2.50	5.00	0.00
Whey powder	30.00	15.00	0.00
Plasma spray-dried	4.00	2.00	0.00
Fish meal	3.00	1.50	0.00
Soy oil	2.00	2.00	0.00
Choice white grease	0.00	0.00	2.50
Dicalcium phosphate 18.5%	1.05	1.05	1.65
Limestone	0.54	0.76	0.50
Salt	0.30	0.30	0.30
Vitamin premix ^5^	0.11	0.11	0.11
Mineral premix ^5^	0.29	0.29	0.29
ZnO, 72% Zn	0.28	0.28	0.00
L-lysine HCl	0.15	0.23	0.34
MHA ^6^	0.04	0.05	0.06
L-threonine	0.00	0.04	0.09
L-tryptophan	0.00	0.00	0.01
Total	100.00	100.00	100.00
Nutrient composition			
ME ^7^, kcal/kg	3478	3454	3404
CP ^7^, %	21.7	21.91	20.46
Fermentable fiber, %	8.51	10.49	12.13
SID ^8^, %			
Lys	1.31	1.28	1.20
Thr	0.79	0.77	0.72
Met	0.32	0.33	0.33
Met+Cys	0.67	0.65	0.61
Trp	0.27	0.25	0.23
Ca, %	0.85	0.79	0.70
Total P, %	0.81	0.71	0.70
STTD ^9^ P, %	0.57	0.44	0.39

^1^ P1-51% represented phase 1 basal diet with standardized ileal digestible (SID) total sulfur amino acid to lysine ratio (TSAA:Lys) at 51%. The phase 1 experimental diets with 58%, 65%, 72%, and 79% SID TSAA:Lys were made by adding 0.10%, 0.21%, 0.31%, and 0.42% MHA in the basal diet, respectively. ^2^ P2-51% represented phase 2 basal diet with standardized ileal digestible (SID) total sulfur amino acid to lysine ratio (TSAA:Lys) at 51%. The phase 2 experimental diets with 58%, 65%, 72%, and 79% SID TSAA:Lys were made by adding 0.10%, 0.21%, 0.32%, and 0.42% MHA in the basal diet, respectively. ^3^ P3-51% represented phase 3 basal diet with standardized ileal digestible (SID) total sulfur amino acid to lysine ratio (TSAA:Lys) at 51%. The phase 3 experimental diets with 58%, 65%, 72%, and 79% SID TSAA:Lys were made by adding 0.11%, 0.20%, 0.31%, and 0.41% MHA in the basal diet, respectively. ^4^ HP300 (Hamlet Protein, Findlay, OH, USA) was produced by treating conventional dehulled soybean meal with a proprietary mixture of enzymes to reduce concentrations of oligosaccharides and anti-nutrient factors. ^5^ Vitamin premix supplied the following nutrients per kilogram of diets: vitamin A, 11,000 IU; vitamin D, 1760 IU; vitamin E, 83.6 IU; vitamin K, 5.5 mg; thiamine, 3.52 mg; riboflavin, 13.2 mg; niacin, 70.4 mg; pantothenic acid, 39.6 mg; pyridoxine, 7.04 mg; folic acid, 1045 µg; biotin, 275 µg; vitamin B12, 55 µg. Mineral premix supplied the following nutrients per kilogram of diet: Zn, 120 mg as zinc sulphate; Cu, 12 mg as copper; Mn, 30 mg as manganese sulphate; Fe, 80 mg as ferrous sulphate; I, 0.4 mg as ethylenediamine dihydriodide; and Se, 0.3 mg as sodium selenite. ^6^ MHA is dry calcium salt of D, L-2-hydroxy-4-(methylthio)butanoic acid (84% Met activity, MHA, Novus International, Inc., St. Charles, MO, USA). ^7^ ME and CP represented metabolizable energy and crude protein, respectively. ^8^ SID = standardized ileal digestible. ^9^ STTD = standardized total tract digestible.

**Table 3:** Effect of antibiotic growth promoter (AGP) and standardized ileal digestible (SID) total sulfur amino acids to lysine ratio (TSAA:Lys) on growth performance of nursery pigs (factorial analysis, Experiment 1).

	AGP		SID TSAA:Lys, %				*p*-Value		
Items	No	Yes	51	58.5	66	SEM	AGP	TSAA	AGP × TSAA
Initial BW, kg	7.9	7.9	7.9	8.0	7.9	0.1	0.85	0.91	0.50
Final BW, kg	16.3	16.7	16.1	16.5	16.8	0.2	0.03	0.03	0.39
ADG, g/d	397	417	391	409	421	9	0.01	0.01	0.39
ADFI, g/d	616	647	616	633	645	12	<0.01	0.06	0.96
G:F, g/g	0.643	0.645	0.634	0.645	0.653	0.009	0.83	0.06	0.08

BW, body weight; ADG, average daily gain; ADFI, average daily feed intake; G:F, gain to feed ratio.

**Table 4:** Effect of increasing standardized ileal digestible total sulfur amino acids to lysine ratios on growth performance of nursery pigs raised without antibiotics (Experiment 2).

	SID TSAA:Lys, %						*p*-Value			
Items	51	58	65	72	79	SEM	Diet	Linear	Quadratic	Cubic
BW, kg										
d 0	5.1	5.1	5.1	5.1	5.1	<.01	0.91	0.85	0.43	0.71
d 7	6.6	6.6	6.6	6.5	6.6	0.1	0.94	0.77	0.60	0.53
d 21	10.5	10.6	10.8	10.8	10.6	0.1	0.30	0.19	0.09	0.76
d 42	21.2	21.7	21.9	21.6	21.9	0.2	0.02	0.09	0.19	0.30
ADG, g/d										
d 0 to 7	211	214	209	208	218	8	0.93	0.77	0.58	0.50
d 7 to 21	278	289	302	302	287	7	0.07	0.15	0.01	0.42
d 21 to 42	511	526	532	516	535	8	0.23	0.33	0.71	0.26
d 0 to 21	256	264	271	271	264	6	0.31	0.19	0.09	0.77
d 0 to 42	384	395	401	394	401	4	0.02	0.01	0.15	0.10
ADFI, g/d										
d 0 to 7	237	240	232	236	235	6	0.94	0.68	0.87	0.80
d 7 to 21	325	351	351	340	335	11	0.45	0.84	0.09	0.39
d 21 to 42	728	718	748	737	721	9	0.14	0.86	0.37	0.15
d 0 to 21	296	314	311	305	301	8	0.54	0.95	0.14	0.39
d 0 to 42	511	514	524	520	511	6	0.40	0.71	0.09	0.50
G:F, g/g										
d 0 to 7	0.889	0.894	0.900	0.880	0.924	0.023	0.73	0.45	0.56	0.39
d 7 to 21	0.837	0.830	0.863	0.900	0.868	0.021	0.16	0.29	0.95	0.08
d 21 to 42	0.707	0.736	0.721	0.702	0.746	0.014	0.18	0.46	0.73	0.08
d 0 to 21	0.846	0.843	0.873	0.896	0.865	0.012	0.03	0.12	0.90	0.09
d 0 to 42	0.754	0.770	0.766	0.758	0.767	0.011	0.85	0.30	0.77	0.21

BW, body weight; ADG, average daily gain; ADFI, average daily feed intake; G:F, gain to feed ratio.

## Data Availability

The data presented in these two studies are available from the corresponding author on request.
